# ASSOCIATION BETWEEN SUBJECTIVE SOCIAL STATUS AND PHYSICAL FRAILTY AMONG OLDER ADULTS IN INDIA

**DOI:** 10.1093/geroni/igad104.0876

**Published:** 2023-12-21

**Authors:** T Muhammad, Manacy Pai

**Affiliations:** International Institute for Population Sciences, Mumbai, Maharashtra, India; Kent State University, Kent, Ohio, United States

## Abstract

Research on the association between subjective social status (SSS) and physical frailty is critical to crafting interventions that can combat functional decline, prolong physical vitality, and sustain quality of life. We assessed the association between SSS and physical frailty and the mediation of this association by perceived everyday discrimination and ill-treatment among older adults in India. Data come from the Longitudinal Aging Study in India with a sample of 31,464 older adults age 60 years and above. Physical frailty was assessed using an adapted version of the frailty phenotype developed by Fried and colleagues. SSS was assessed using the Macarthur scale. Multivariable logistic regression along with KHB (Karlson–Holm–Breen) method was employed to examine the associations and the mediation effects. The prevalence of physical frailty was 30.65% and those with lowest SSS had higher prevalence of physical frailty (42.06%). After adjusting for a number of confounders, odds of physical frailty were significantly lower among those with a high SSS in comparison to those with low SSS, and the variance explained by the SSS was higher than that explained by household consumption quintiles. The association between SSS and physical frailty was mediated by perceived everyday discrimination (percent mediated: 8.20) and experiences of ill-treatment (percent mediated: 9.32). The findings underscore that when examining the association between socioeconomic status and physical frailty, it is important to consider SSS given that perceptions of one’s social standing likely reflect the less apparent psychosocial components associated with it.

